# Identical Functional Organization of Nonpolytene and Polytene Chromosomes in *Drosophila melanogaster*

**DOI:** 10.1371/journal.pone.0025960

**Published:** 2011-10-11

**Authors:** Tatyana Yu. Vatolina, Lidiya V. Boldyreva, Olga V. Demakova, Sergey A. Demakov, Elena B. Kokoza, Valeriy F. Semeshin, Vladimir N. Babenko, Fedor P. Goncharov, Elena S. Belyaeva, Igor F. Zhimulev

**Affiliations:** Institute of Molecular and Cellular Biology of the Siberian Branch of the Russian Academy of Sciences, Novosibirsk, Russia; University of Dayton, United States of America

## Abstract

Salivary gland polytene chromosomes demonstrate banding pattern, genetic meaning of which is an enigma for decades. Till now it is not known how to mark the band/interband borders on physical map of DNA and structures of polytene chromosomes are not characterized in molecular and genetic terms. It is not known either similar banding pattern exists in chromosomes of regular diploid mitotically dividing nonpolytene cells. Using the newly developed approach permitting to identify the interband material and localization data of interband-specific proteins from modENCODE and other genome-wide projects, we identify physical limits of bands and interbands in small cytological region 9F13-10B3 of the X chromosome in *D. melanogaster*, as well as characterize their general molecular features. Our results suggests that the polytene and interphase cell line chromosomes have practically the same patterns of bands and interbands reflecting, probably, the basic principle of interphase chromosome organization. Two types of bands have been described in chromosomes, early and late-replicating, which differ in many aspects of their protein and genetic content. As appeared, origin recognition complexes are located almost totally in the interbands of chromosomes.

## Introduction

*Drosophila* salivary gland polytene chromosomes are routinely used as a model for actively functioning interphase eukaryotic chromosomes. Aside from their giant size, polytene chromosomes display prominent banding pattern, which is formed by tight alignment of homologous chromomeres, thereby forming a cable of chromatids with stripes of condensed material. Two neighboring chromomeres are separated by an interchromomeric region, which appears as an interband in the context of a polytene chromosome. According to the early estimates, most of the DNA in chromosomes (95%), and so most of the genes, are found in bands [1], [2]. Genetic composition of interbands still remains mysterious [3]. Despite the availability of *Drosophila* genome, methods to even approximately map the band/interband borders on a physical map are still lacking. Only two polytene chromosome bands – 10A1–2 and 75C1-2 [4], [5] - have been relatively well mapped (at a 5 kb resolution). At the same time, no interbands with exactly mapped borders and DNA sequences are presently known.

As more and more data become available for the features of chromatin organization in different structures of a chromosome, these can be used to demarcate particular chromatin regions on a physical map of genome. For instance, it is widely accepted that at the level of polytene chromosome cytology, many proteins and protein complexes map to the interband regions. In particular, this applies to interband-specific proteins Z4 and Chriz (CHRO) [6]–[8], insulator protein BEAF-32 [9], [10], topoisomerase I [11], “open chromatin”- and transcription-associated proteins, such as actively elongating and initiating RNA polymerase II, P-TEFb, TFIIH, TFIIF, SPT4, SPT5, SPT6, FACT, dMediator, GAF, TRX [12]–[26], nucleoporins [27]. Likewise, interbands are the home base for many nucleosome-remodeling and histone-modifying enzymes, such as WDS highly conservative protein [28]–[30] and NURF, which increases accessibility of chromatin templates [31]. They harbor histone variants H2A2, H2AZ, H3K14ac, H3K9ac – K14Ac, H3K4me3 [32]–[35], proteins of chromatin modulating complexes – CHD1 [36], JIL-1, [37], [38], BRM [39], cohesin [40]. Several DNase hypersensitive sites (DHSs) were discovered in the interband 3C6/C7, which locates in the 5′-regulatory region of *Notch* gene [41]. The *fa^swb^* deletion, which removes these DHSes, leads to the disappearance of this interband [42], [43] (more details and references in [3].

Yet, according to early estimates, interbands account for less than 5% of genomic DNA, i.e. on average one interband corresponds to about 2 kb [1]. Taking into account that nucleosome packaging of interband material further reduces the linear size of these structures, the existing immunostaining methods fail to provide enough resolution in mapping the above-mentioned proteins to faint bands, band/interband borders, interbands or within all these structures.

Earlier we developed an approach to simultaneously map the interband DNA on both physical and cytological maps of polytene chromosomes [44]. This approach took advantage of the fact that when a P-element based transgene integrates into the genome, and if this integration hits an interband region, the transgene forms a new polytene chromosome band that can be clearly visualized at the level of electron-microscopy. Thus, by comparing the physical and cytological maps, one can accurately annotate the sequences adjacent to the integration site of the transpozon as forming an interband. One of the obvious drawbacks of this approach is that it only allows mapping the interband sequences around transgene insertion site, but does not tell us where the band/interband border is; in other words, it fails to provide data that would help characterize the interband as a structure.

More recently, several projects have produced a wealth of information about genome-wide localization patterns of proteins and protein complexes in *Drosophila* cell lines [45]–[49]. When we compared these data with 13 chromosome regions that had EM-mapped transgene insertions in interbands, we observed that such regions were associated with interband-specific proteins such as Chriz/CHRO and other “open chromatin” proteins described above. On a physical map, these interband regions typically corresponded to the intergenic regions and 5′-ends of genes, they were associated with ORCs, RNA polymerase II, transcription factors, nucleosome remodeling factor WDS. Additionally, these interbands showed yet another feature of the open chromatin: increased frequency of P-element insertions. Furthermore, these proteins tend to co-localize with each other [50].

In polytene chromosomes, there are several types of bands. First, there are large densely-condensed and late-replicating bands corresponding to the intercalary heterochromatin (IH) [51]. They are bound by SUUR protein, which marks late-replicating regions of the genome and contributes to the phenomenon of underreplication in polytene chromosomes. In these polytene chromosome bands, the DNA strands frequently fail to complete replication and so form chromosome breaks [52], [53]. Such IH regions are characterized by lower than the genome-average gene density [54].

Clearly then, differences in gene density, replication timing, associated protein factors observed in interbands, dense IH bands and faint decompacted bands – can serve as convenient marks to precisely map the molecular borders of these structures. Combined with EM analysis, these data allow for the first time to compile an accurate molecular and cytogenetic map of bands and interbands in *D. melanogaster* salivary gland polytene chromosomes as well as in chromosomes of the cell lines.

Here, we perform analysis of bands and interbands in the region 10A1–2 – 10B1–2, very well characterized cytology- and genetics-wise before [4], [55], in polytene chromosomes from salivary gland cells and interphase chromosomes of mitotically dividing cells. Our results demonstrate high similarity of the banding patterns in both polytene and non-polytene chromosomes.

## Results

### Electron microscopy analysis of banding pattern in the region 9F13-10B3 of polytene chromosomes

As it is known, Electron Microscopic (EM) sections of polytene chromosomes in which all the known bands in given regions are expected to be seen, occur extremely infrequently. In the majority of sections a complete set of bands is observed only in short chromosome segments, especially in the regions of thin bands. Therefore, several sections of the same chromosome region should be used for analysis of bands (see [2] for more details]).

**Figure 1:**
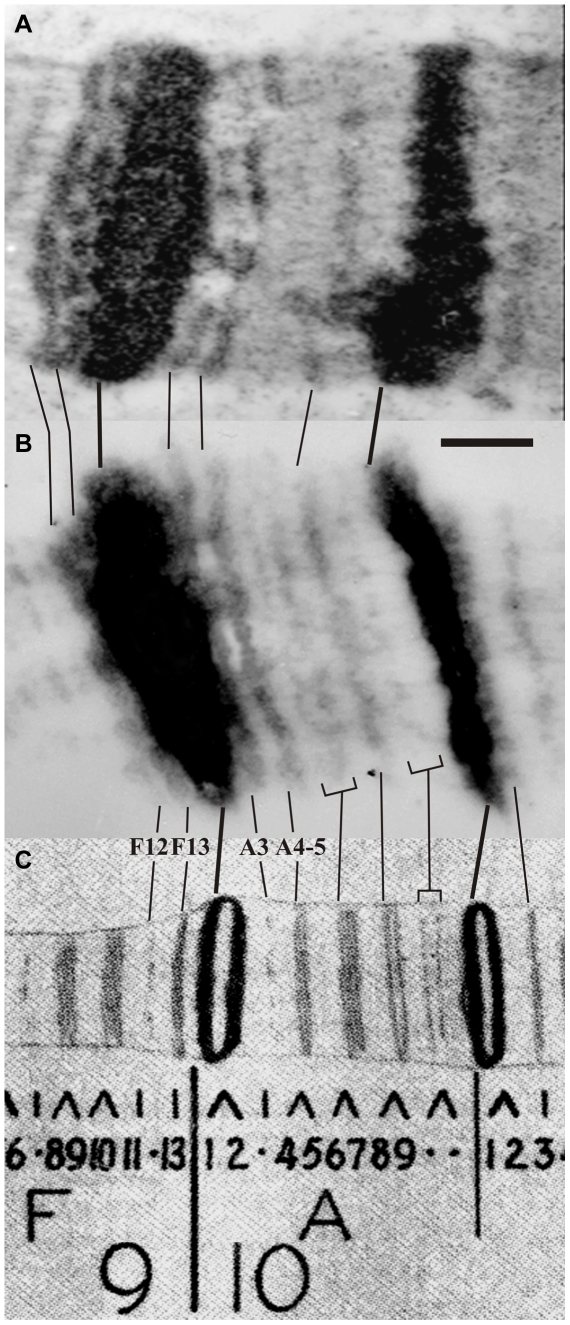
Comparison of several Electron Microscope sections of the region 9F11-12 – 10B (A, B) with revised Bridges map [**56**] **(C).** Vertical lines connect homologous bands. Scale represents 1 µm.

The electron miscopy data we obtained for the regions 10A–B (Fig. 1A, B) are consistent with revised map of Bridges [56] (Fig. 1C), if one considers double bands in the Bridges maps as singlets (see [2], for discussion). Figure 1 A, B illustrates three distinct types of domains characteristic of the banding pattern. First, these are two fairly massive bands 10A1–2 and 10B1–2, encompassing dense chromatin and each flanked from both sides with interbands (with the band 10A2-1 appearing larger than the 10B1–2). In between these two large bands, one can see a «paling» of thin loosely-compacted “grey” bands and interbands, which correspond to the region 10A3–11.

Under the light microscope, in the context of a normal X chromosome, the band 10A1–2 appears as a “single large dense body even if the chromosome is well-stretched” [57], [58], however according to the EM data [4], [55] this region in fact harbors five distinct bands. There is one prominent band 10A1–2, and four medium-sized bands, two found distal (9F12 and 9F13, Fig. 1A) and two situated proximal (10A2 and 10A4–5, Fig. 1A, B). Of these four, two bands are located on the sides of the group – 9F12 and 10A4–5, - can sometimes be seen under the light microscope as separate bands, whereas 9F13 and 10A3 are located too close to 10A1–2 and are virtually never seen as separate bodies. The band 10A4–5 appears as the largest among the paling of the 10A3–A11 group of bands. The next two thin bands at 10A6 and 10A7 are rarely detected even at the EM level, and even less frequently seen separately from each other. Notably, when these bands do appear separate, the 10A7 band looks larger than the 10A6 (Fig. 1B). The band 10A8–9 is routinely seen even under the light microscope, whereas the band 10A10–11 represents a typical singular faint band.

### Mapping of bands and interbands in the 9F13-10B13 region of nonpolytene chromosomes

According to the combined cytogenetic and molecular-genetic analysis, the region of polytene chromosome band 10A1–2 is marked with two genes, *vermilion* which is located at its distal end [55], [57], [58], and *sevenless*, at its proximal side [4], [55]. Therefore, we used positions of these two genes on the physical map as starting points for mapping the region in nonpolytene chromosomes.

**Figure 2:**
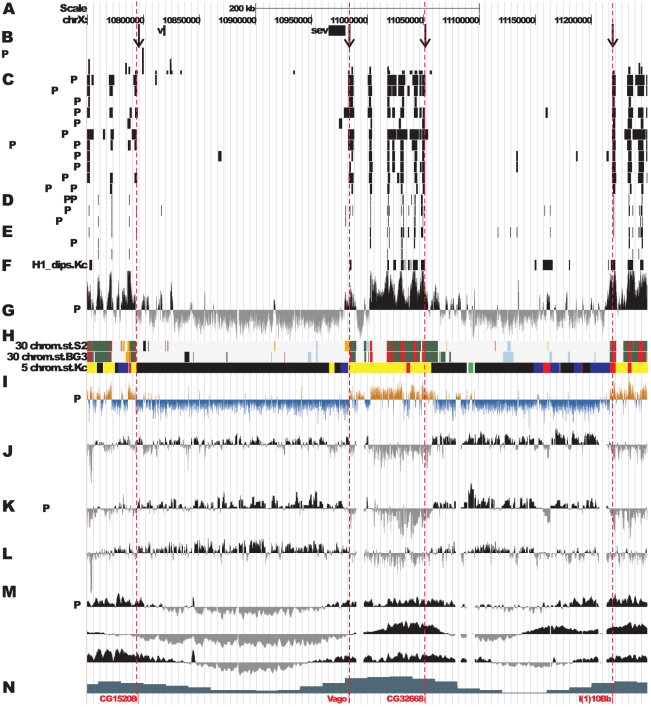
Localization of proteins and DNA elements in 9F13 – 10B3 region of nonpolytene chromosomes (according to data of modENCODE). The positions of proteins were located as described in Material and Methods. **A** - physical map of DNA; positions of *v* and *sev* genes are taken from FlyBase, arrows 1–4 indicate position of probes for FISH on physical map. **B** - *P*-elements density in the region calculated as number of insertions per 1 kb in 10 kb interval (data on insertions are taken from FlyBase) **C** - interband specific and active chromatin specific proteins in S2 cells [48], **D** - DNase I hypersensitivity sites (DHS) in S2, BG3 and Kc cells [48] **E** - ORC2-binding sites in S2, BG3 and Kc cultural and salivary gland cells [59] **F** - histone H1 dips localization in Kc cells [45] **G** - histone H3.3 localization in S2 cells (modENCODE, Henikoff group) **H** - 30 chromatin states in BG3 and S2 cells [48], and 5 chromatin types in Kc cells [47] **I** - nucleosome turnover dynamics in S2 cells [60], **J** - D1 localization in Kc cells [47], **K** - SUUR localization in Kc cells [47], **L** - Lamin localization in Kc cells [47] **M** - early (up) and late (down) replication in S2, Kc and BG3 cells [61] **N** - gene density (number of genes per 10 kb of DNA) [54].

Figure 2 shows map of the DNA features and protein localization profiles in the region of interest in nonpolytene chromosomes; these data have been generated by fly modENCODE consortium [47]–[49], [59]–[62] as well as in experiments of genome-wide analysis of chromosome proteins [47].

Much like as was observed with the EM mapping data of polytene chromosomes, one can subdivide this region into three distinct domains. First, there are two zones on both flanks of the region (Fig. 2), which were previously shown to lack any interband-associated proteins. The DNA sequences that map to these zones are very large and are flanked with regions displaying interband-like features (Fig. 2C, D, E, F, G). According to the physical map, the leftmost domain may correspond to the 10A1–2 band, as it comprises *vermilion* and *sevenless* genes (Fig. 2A). The other region showing similar properties should correspond to the large band 10B1–2. Mimicking the EM pattern observed in polytene chromosomes, the region in between 10A1–2 and 10B1–2 appears as a paling of alternating interband-like zones and regions lacking interband features (middle of the Fig. 2C, D, E, F, G, H). This region is composed of faint bands intermingled with interbands which corresponds well to the EM mapping data (see Fig. 1).

So, in nonpolytene chromosomes of mitoticaly dividing cells one can find DNA fragments demonstrating features characteristic to polytene chromosomes, i.e. bands and interbands.

### Identical positions of bands and interbands in polytene and nonpolytene chromosomes on genome map

We asked whether interbands have the same borders in nonpolytene and polytene chromosomes. To do so, we first selected two interband fragments on both sides of the 10A1–2 and 10B1–2 bands of nonpolytene chromosomes. The large sizes of both bands allow accurate mapping of said DNA fragments using FISH and immunostaining.

**Figure 3:**
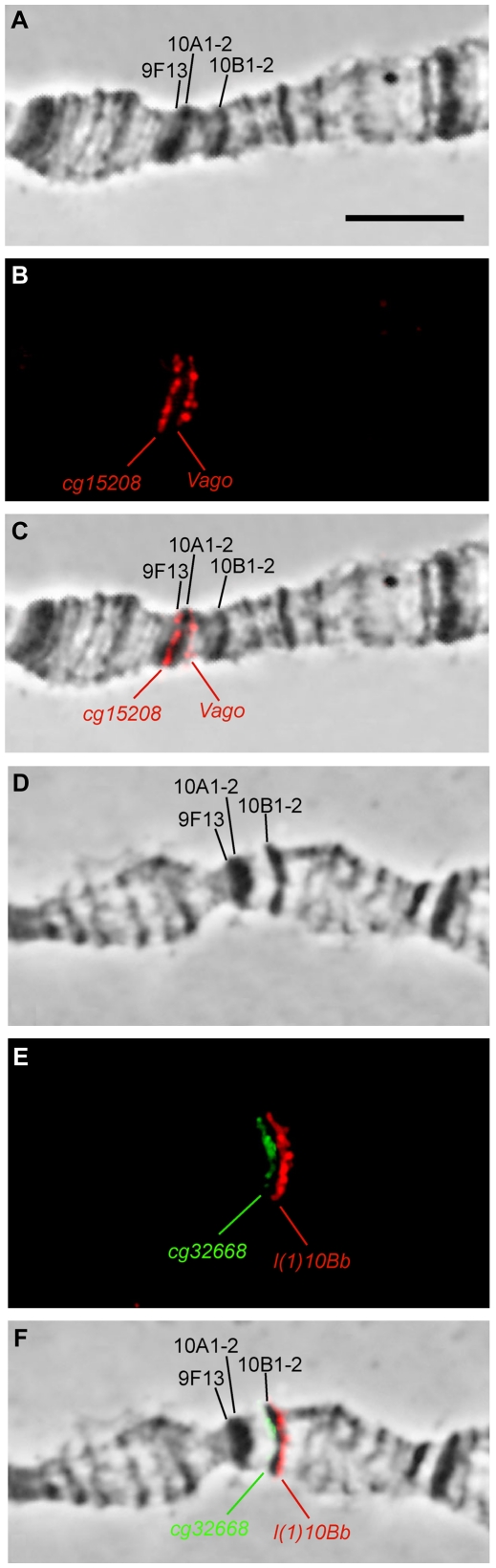
Localization in polytene chromosomes of DNA fragments, which according to modEncode data are located in the predicted interbands flanking the 10A1–2 and 10B1–2 bands of the nonpolytene chromosomes. Banding pattern in the region under phase contrast microscope (**A, D**), pairwise FISH mapping of DNA fragments, limiting the bands 10A1–2 (probes CG15208 and Vago) (**B, C**) and 10B1–2 (CG32668 and l(1)10Bb) (**E, F**).

A short chromosome fragment with interband feature which is found immediately distal to the tentative 10A1–2 in nonpolytene chromosomes (arrow 1 on Fig. 2A) has been mapped on polytene chromosomes using three DNA probes (Table 3 in Materials and Methods section): *CG1582*, *spas 9F* and *CG15208* (hereafter the names of FISH probes correspond to the gene names that they map to). Given that the 10A1–2 band frequently fuses with the neighboring distal 9F11-12 and 9F13 bands (Fig. 3A), we only analyzed rare polytene chromosome spreads where all the bands appeared separate from each other. The three above-mentioned probes were mapped to the interval between the faint band 9F13 and the distal edge of 10A1–2 (Fig. 3B, data shown for *CG15208* only), i.e. in the expected interband 9F13/10A1–2.

The region found on proximal side of the 10A1–2 band of nonpolytene chromosomes, has been mapped using two DNA probes, *Vago* and *CG2076* (arrow 2 on Fig. 2A). The signal maps to the proximal edge of 10A1–2 in polytene chromosomes (Figs. 3B, C show the data for *Vago* probe only), i.e. to the interband 10A1–2/10A3.

The *CG32668* and *l(1)10Bb* genes (arrows 3 and 4 on Fig. 2A) located distally and proximally to the tentative 10B1–2 band of the nonpolytene chromosomes, map by FISH to the interbands flanking this band in polytene chromosomes (Figs. 3 D, E, F). So, the four probes essentially hybridize to interband regions immediately flanking the two dark bands of the region 10A–B.

**Figure 4:**
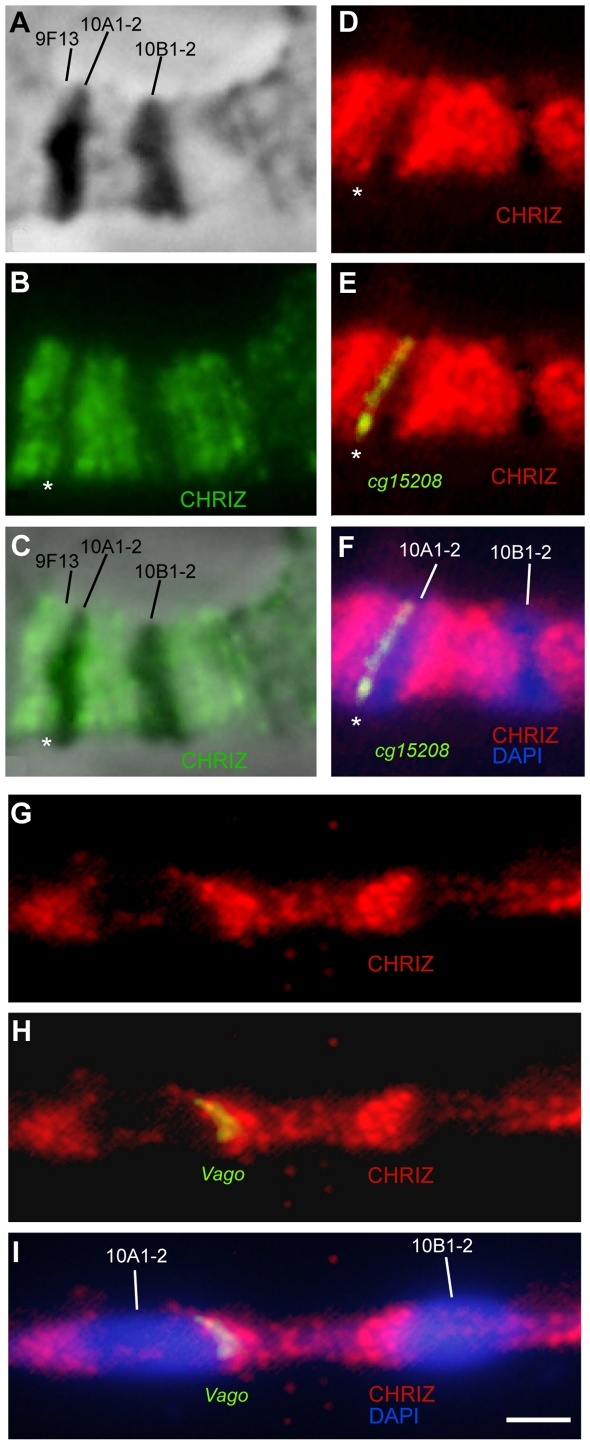
Colocalization of DNA probes limiting the 10A1–2 band with the Chriz/CHRO protein. Localization of the Chriz protein in the 10A–B region (**A–C**); the bands in the region under phase contrast (**A**), the Chriz/CHRO protein, asterisk points to the interband 9F13/10A1–2 B), match of Chriz/CHRO location and phase contrast (**c**), colocalization of the DNA probe CG15208, limiting distal side of the band 10A1–2 and Chriz/CHRO protein (**D–F**); colocalization of the DNA probe Vago, limiting proximal side of the band 10A1–2 and Chriz/CHRO protein on stretched chromosomes (**G–I**). Bar represents 2 µm.

To address the question of whether Chriz/CHRO-associated DNA fragments are the same in polytene and nonpolytene chromosomes, we performed simultaneous FISH for these interband sequences and immunodetection of Chriz/CHRO in polytene chromosomes. Figure 4A, B, C shows that there is only one Chriz/CHRO-positive region between the bands 9F13 and 10A1–2 (marked with an asterisk on Fig. 4B, C, D, E, F), i.e. Chriz/CHRO mapped in the 9F13/10A1–2 interband in nonpolytene chromosomes does map to the 9F13/10A1–2 interband in polytene chromosomes. At the same time, the FISH probe of *CG15208* which bind this protein in nonpolytene chromosomes (Fig. 2) displays perfect co-localization with Chriz/CHRO in this region of polytene chromosomes (Fig. 4D, E, F).

**Figure 5:**
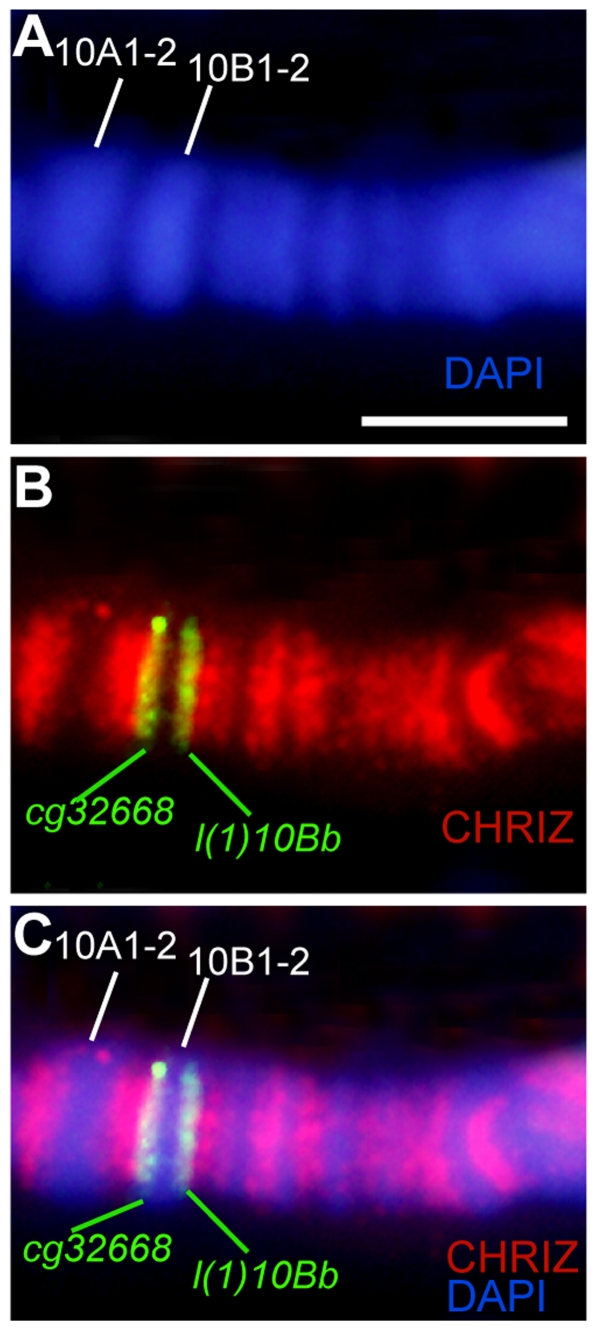
Colocalization of DNA probes limiting the band 10B1–2, and Chriz/CHRO protein. Banding pattern in the region 10A–B (DAPI) (**A**), immunostaining of Chriz/CHRO and FISH of the DNA probe (**A**), immunostaining of Chriz/CHRO, FISH of the DNA probe and DAPI (**C**). Bar represents 5 µm.

We successfully mapped *Vago* probe on stretched polytene chromosomes which, as accepted [2, for more details], provided greater resolution. Figures 4G, H demonstrate that upon stretching, both the length of large bands and their spacing have dramatically increased, which, in turn, resulted in that Chriz binding pattern in between 10A1–2 and 10B1–2 is now seen as comprising a series of relatively distinct fluorescent bands reminiscent of the EM map for this region (Fig. 4I). We also observed that, first, *Vago* probe located in interband of the nonpolytene chromosome also completely co-localizes with one of these Chriz/CHRO -positive regions (Fig. 4G, H, I), and this probe itself mapped to the same interband in the proximal edge of the 10A1–2 band of polytene chromosome. Similarly, *CG32668* and *l(1)10Bb* that in nonpolytene chromosomes flanked 10B1–2 from both sides, displayed extensive co-localization with Chriz/CHRO -bound fragments in the 10B1–2 band in polytene chromosomes (Fig. 5).

Taking into account that Chriz/CHRO is a protein found both in the “open” chromatin of nonpolytene chromosomes and in polytene chromosome interbands tagged with P-element insertions [50], we conclude that DNA sequences associated to Chriz/CHRO proteins correspond to interbands of polytene chromosomes. This, in turn, indicates that Chriz/CHRO is invariably bound to interbands in chromosomes of various cell types. Thus, we can map all the interband borders in a given region of a physical map, using the localization borders of various interband-specific proteins (Fig. 2C, D, E, F, G).

### Mapping the band/interband borders on physical map and molecular-genetic characterization of bands and interbands

In order to determine the span of DNA sequences in each interband in this region, we first plotted the localization profiles for proteins on nonpolytene chromosomes and those we previously selected as markers for the transposon-tagged interbands of polytene chromosomes: this list included Chriz/CHRO, WDS, ORC, BEAF as well as DNase I hypersensitive sites and histone H1 density dips (see Fig. 2). Using these data, we identified the limits of interbands (Table 1). Within the region of interest, there are nine such interband regions, which is exactly the number of interbands on the Bridges [56] and EM (see Fig. 1) maps. Table 1 shows the coordinates of interband borders and the length of interband DNAs. Distances between interband borders, thus, correspond to the lengths of bands, totaling eight (in agreement with both Bridges and EM maps. Two out of these bands can be classified as late replicating bands, while the rest six are regular early-replicating (Table 1). The chromosome region from 9F13 to 10B3 encompasses 428,307 bp, nine interbands account for 20,100 bp, i.e. 4.69% of DNA length. Interbands range from 1,336 to 4,181 bp in size, with average size about 2,233 bp.

**Table 1:** Coordinates and sizes of bands and interbands on physical map of the 9F13 – 10B3 region.

Cytological region	Position in genome (in nucleotides)	Length (in nucleotides)
9F13/10A1–2^IB^	10792800..10793400	600
10A1–2^B^	10793401..10984199	190798
10A1–2/10A3^IB^	10984200..10985600	1400
10A3^B^	10985601..11002399	16798
10A3/10A4–5^IB^	11002400..11004400	2000
10A4–5^B^	11004401..11017399	12998
10A4–5/10A6^IB^	11017400..11020400	3000
10A6^B^	11020401..11022399	1998
10A6/10A7^IB^	11022400..11024000	1600
10A7^B^	11024001..11029999	5998
10A7/10A8–9^IB^	11030000..11033000	3000
10A8–9^B^	11033001..11041199	8198
10A8–9/10A10^IB^	11041200..11044000	2800
10A10–11^B^	11044001..11048599	4598
10A10–11/10B1–2^IB^	11048600..11050000	1400
10B1–2^B^	11050001..11217799	167798
10B1–2/10B3^IB^	11217800..11220400	2600

Notes: IB-interband; B-band.

Data of release FB2011_03 were used.

Lengths of DNA sequences mapping within bands vary in much broader range. Eight bands concerned here account for 408,207 bp DNA, i.e. average length of DNA per band is roughly 51 kb. The largest IH bands 10A1–2 and 10B1–2, span 189 kb and 170 kb, respectively, whereas the smallest bands from around the 10A6 region are as short as 2761 bp, which is about 69 times less than found in the largest band 10A1–2. The region of faint bands at 10A3–10A11 encompasses about 52 kb DNA, and these bands show much more constrained variation, from 2761 to 14634 bp, being 8725 bp on average.

Accurate mapping of band and interband borders on the physical map and proper identification of the band/interband material, allows us to describe and characterize two types of polytene chromosome bands: late- and early-replicating bands, and give new insight into the structure of interbands.

Late-replicating IH bands 10A1–2 and 10B1–2 composed of compacted material, as is seen by cytology [51]. Degree of DNA compaction in them, i.e. the ratio of visual length of the bands to their actual DNA length, is highest (158- and 204-fold compaction, respectively) (Table 2). These bands are late replicated in both polytene [51] and nonpolytene [61] chromosomes (Fig. 2N).

**Table 2:** DNA compaction ratio of the bands in the 9F13 -10B3 region.

Bands and interbands (IB)	Length along chromosome axis^a^ (mcM) (*a*)	Physical sizes (bp) (*b*)	Length of DNA (µm) (*c* = *b*×0,34)	Compaction ratio (*c/a*)
9F13/10A1–2^IB^	0,043±0,009	600	0,20	4,65
10A1–2^B^	0,410±0,046	190798	64,87	158,22
10A1–2/10A3^IB^	0,095±0,019	1400	0,48	5,05
10A3^B^	0,078±0,006	16798	5,71	73,21
10A3/10A4–5^IB^	0,065±0,008	2000	0,68	10,46
10A4–5^B^	0,092±0,007	12998	4,42	48,04
10A4–5/10A6^IB^	0,144±0,023	3000	1,02	7,08
10A6^B^	0,057±0,006	1998	0,68	11,93
10A6/10A7^IB^	0,045±0,008	1600	0,54	12,00
10A7^B^	0,064±0,005	5998	2,04	31,87
10A7/10A8–9^IB^	0,144±0,006	3000	1,02	7,08
10A8–9^B^	0,087±0,003	8198	2,79	32,07
10A8–9/10A10^IB^	0,117±0,006	2800	0,95	8,12
10A10–11^B^	0,073±0,003	4598	1,56	21,37
10A10–11/10B1–2^IB^	0,0975±0,0055	1400	0,48	4,92
10B1–2^B^	0,279±0,011	167798	57,05	204,48
10B1–2/10B3^IB^	0,086±0,006	2600	0,88	10,23

a – estimated on electron microscope sections of 50 polytene chromosomes.

Notes: IB-interband; B-band.

Data of release FB2011_03 were used.

The DNA in such bands displays several common features, such as noticeably decreased level of ORC2 localization (Fig. 2E), complete absence of any of the “open chromatin” ensemble of proteins which are normally found in interbands (Chriz/CHRO, BEAF-32, RNA polymerase II, BRE-1, WDS, NURF, TRX) (Fig. 2C, D, E, F, G), and low frequency of intergration of P-element based transgenes, insertions of which are also characteristic for open chromatin (Fig. 2B).

Recently, by integrative analysis of genome-wide binding maps 53 broadly selected chromatin components in *Drosophila* cells it was shown that the genome is segmented into five principal chromatin types that are defined by unique combinations of proteins and form specific domains. Each of these chromatins were conditionally labeled with a color: BLUE and BLACK – repressive chromatins, RED and YELLOW – transcriptionally active chromatins, GREEN – heterochromatic domain (see Filion et al., 2010 [47] for details and protein compositions of each domains). In other work the genome-wide chromatin landscape based mainly on eighteen histone modifications and several non-histone chromatin proteins was summarized by 30 combinatorial patterns or states [48].

Bands like 10A1–2 and 10B1–2 can be categorized as having the chromatin state 30, which is described as lacking any active chromatin marks in the fly modENCODE [48]. In both 10A1–2 and 10B1–2 bands, strong enrichment for SUUR, D1 and lamin B is observed [47], [63] (Fig. 2L, M) which is characteristic for BLACK chromatin, with depletion for H3.3 (Fig. 2G) and low level of newly synthesized histone subunits (Fig. 2J). These regions show low gene density, which is characteristic of late replicating regions of the genome [54], [64] (Fig. 2O), they show no depletion for histone H1, which is necessary for higher-order nucleosome packaging (Fig. 2F).

Yet, these bands display several distinct features. The band 10A1–2 is virtually homogeneous in terms of its principal chromatin “color” - it is BLACK [47] throughout, showing pronounced enrichment in SUUR, lamin B, and D1 proteins. Only its proximal-most part shows some contribution of BLUE and YELLOW chromatins (Fig. 2I).

The band 10B1–2 appears as a more complex body. In both polytene and nonpolytene chromosomes, this band always appears as a single unit, flanked by interbands from both sides. However, depending on the differentiation stage, the chromatin state within 10B1–2 can change. For instance, in diploid cells, the RED chromatin typical of interbands is present, or some features of chromatin state #1 become apparent (Fig. 2H). Despite the fact that overall gene density throughout 10B1–2 is decreased, the corresponding late completion of replication and SUUR binding in Kc cells is only observed in the distal 40% of this band (Fig. 2N, O). In Kc cells, Lamin B is found associated with both a fraction of late-replicating sequences and all of the early-replicating sequences of 10B1–2 (Fig. 2K, L, M, N). Notably, one of the bands - 10A1–2 – has all its genes replicating late, whereas only a fraction of genes from within 10B1–2 appears late-replicating (Fig. 2N). In this latter case, one can view the 10B1–2 band as only partially composed of late-replicating material.

And, finally, the band 10B1–2 is mosaic in terms of “colored” chromatin types [47]. Even though it is mostly composed of BLACK and BLUE chromatins, it also encompasses YELLOW chromatin at its distal edge, which correlates with localization of histone H1 dips (Fig. 2I).

Very thin (ca 9 kb/band on average, see above) early-replicating bands are harbored in the region 10A3–10A11 (Figs. 1 and 2). These bands are distinct from IH bands in many ways. They complete replication early, do not contain BLACK or BLUE chromatin, however, and much like the IH bands, they show poor enrichment (if any) for ORC2 (Fig. 2D, E). Morphology-wise, they appear less dense, the degree of DNA compaction in them was found to be 16–38 fold, which is much higher than in interbands, but lower than in IH bands. Notably, two bands, 10A3 and 10A4–5, that are adjacent to 10A1–2 display the level of compaction somewhat higher, around 54–63 fold (Table 2). These bands do not associate with RNA polymerase II, interband-specific proteins, although they are composed of YELLOW, i.e. transcriptionally-active, chromatin [47]; according to [48] they contain chromatin states ## 22–24 and 30. It must be emphasized that these bands lack DNase I hypersensitive sites.

Interbands. appear decondensed in terms of their morphology. The minimal DNA compaction according to data of Table 2, is observed in interbands (3- to 15- fold. Additionally, they display major features of “open” chromatin, such as DNase I hypersensitive sites (DHS), interband-specific proteins Chriz/CHRO, BEAF, PolII, various transcription factors, histone variant H3.3, nucleosome-remodelling factors such as WDS and BRE-1, histone H1 dips. In all interbands, the chromatin is YELLOW or RED (see Fig. 2I), which is indicative of their participation in transcriptional activity [47].

**Figure 6:**
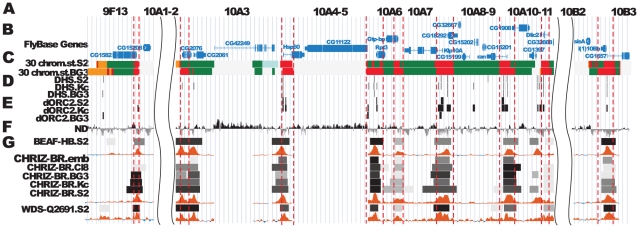
Relation of genetic map, and band/interband pattern in the region 9F13 – 10B3. **A** - predicted bands **B** - FlyBase genes **C** – 30 chromatin states in BG3 and S2 cells [48] **D** - DNase I hypersensitivity sites (DHS) in S2, BG3 and Kc cells [48] **E** - ORC-binding sites in S2, BG3 and Kc cells [59] **F** – Nucleosome Density (modENCODE, Henikoff group) **G** - active chromatin specific [48] and - interbands specific proteins. Predicted interbands (dotted vertical lines are according to peaks in distribution of corresponding elements, solid lines reflect the edges of distributions of different characteristics).

According to the 30 chromatin state model [48], interbands fall into states #1–6. These are exactly the same properties that we previously found specific for 13 interbands mapped throughout the analysis of P-transgene insertions [50]. In that work we showed, that 11 of the 13 transgene-tagged interbands corresponded to either intergenic regions or 5′-ends of genes. Moreover, chromatin state #1 is enriched in TSSes, 5′UTRs and start codons [48]. As the Figure 6 demonstrates the comparison of gene localization from FlyBase with chromatin state #1 [48], interbands/bands, enrichment profiles for Chriz/CHRO, WDS, ORC2 and a nucleosome density plot. The dashed lines that mark the 9F13/10A1–2 interband, maps to the intergenic region between the 5′-ends of *CG1582* and *CG15208*. The interband 10A1–2/10A3 comprises the 5′-half of the *CG2076* gene (see Flybase). The interband 10A3/10A4–5 maps to the intergenic region between *CG42249* and *Hsp60*. Likewise, interband 10A4–5/10A6 is found between divergently transcribed *CG11122* and *Rpt3*. The central part of the 10A6/10A67 interband corresponds to the 5′-end of *Gtp-bp*; center of 10A7/10A8–9 interband maps to the 5′-end of *Klp10A* and *CG18292*, the interband 10A8–9/10A10–11 corresponds to the common upstream regulatory regions for *ran* and *CG1908*. The 5′-end of *Dlic2* makes up the 10A10–11/10B1–2 interband, whereas 10B1–2/10B3 interband occupies the 5′-ends of *l(1)10Bb* and *CG1657* (Fig. 6). Thus, of the nine interbands studied in this work, eight map to the 5′-ends of genes, intergenic regions or first exons of genes.

DNA sequences that bind ORC2 in the nonpolytene chromosomes are unevenly distributed along this region [49]. For example, the band 10A1–2 lacks any ORC2 binding (Fig. 2E), whereas 10B1–2 shows four regions or ORC2 enrichment in Kc cells, but not in other cell types (Fig. 2E). ORC2 appeared virtually uniformly present throughout the region 10A3–10A11, with all origin recognition complexes being invariably found in interbands (Figs. 2 and 6). For instance, this 68 kb-long region (between 10A1–2 and 10B1–2 bands) encompasses 5–7 ORC2 binding sites(13.6 – 9.7 kb per ORC2), whereas 10A1–2 region shows no ORC2 binding over 190 kb, in the 10B1–2 band the ORC2 density varies from 42.5 kb/ORC2 to complete absence in 168 kb in chromosomes of some cell lines.

## Discussion

The major conclusion that can be drawn from the present work is that both polytene and non-polytene chromosomes of mitotically active cells display band/interband organization. Furthermore, localization and protein content of interband chromatin in these types of interphase chromosomes are identical. This conclusion is based on the following two groups of facts.

Previously, we studied organization of interbands in diploid cells by accurately mapping P-element-tagged interbands in the genome [50]. Analysis of thirteen interband regions thus mapped demonstrated their identical organization in polytene and non-polytene chromosomes. More detailed analysis of two of these interbands firmly established that “interband as a stretch open chromatin is conserved in structure … between cell lines” [65]. In the present work, we used a reverse approach. We first mapped the interbands in the region 9F13-10B3 based on the localization of interband-enriched chromatin features. Then, using FISH with DNA probes from interband regions, we observed that interband positions in diploid cells matched those found in polytene chromosomes. This conclusion is also supported by perfect co-localization of interband DNA and interband-specific protein CHRIZ.

Based on these data, we hypothesized that interbands could represent a basic unit of interphase chromosome organization across different cell types. This conclusion is consistent with earlier observations of conserved banding pattern in different larval and adult cells in many dipteran species (see [1] and [66] for more details and references). Importantly, it must be noted that those cytological observations were indeed limited to tissues with polytene chromosomes. We generalize this conclusion as applied to the chromosomes from diploid non-polytene cells. Conserved localization and organization of interbands thus argues in favor of their important functions in the chromosome.

Presently, interbands' functions remain enigmatic. In this respect, it would be of utmost interest to explore the mechanisms of binding, roles and interplay between interband-specific proteins, such as CHRIZ, Z4 and JIL1, which were previously shown to contribute to the maintenance of open chromatin structure in interbands [6], [38]. It has recently been demonstrated that CHRIZ and Z4 form a complex required to recruit JIL1 kinase thereby enabling H3S10 phosphorylation of histones in interband nucleosomes. This might in turn result in chromatin decondensation in interbands [65].

Open chromatin is required for binding of pre-replication machinery components [49]. So, given that interbands are particularly rich in ORC2, they might have a special role in replication initiation.

It is also important to consider particular properties of interband DNA organization. At the level of DNA sequence, interbands are poorly conserved; they typically correspond to intergenic, 5′-regulatory and 5′-untranstlated regions of genes. Notably, not all interband material is functionally equivalent. For instance, DNaseI hypersensitive sites (DHSs) and histone H1 dips were particularly enriched in interbands [50]. Several DHSs were discovered in an interband 3C6/C7, which maps to the 5′-regulatory region of the *Notch* gene. The *fa^swb^* deletion which removes 900 b.p. including these DHSs, leads to the disappearance of this interband [41]–[43]. Removal of just 246 b.p. closest to the transcription initiation site from within these 900 b.p. and containing just one DHS results in the compaction of the rest of the interband sequence. So, the DNaseI hypersensitive site in the interband 3C6/7 is important for the interband formation [67]. It thus appears highly promising to further analyze interband DNA in order to dissect its functionally important sites.

**Figure 7:**
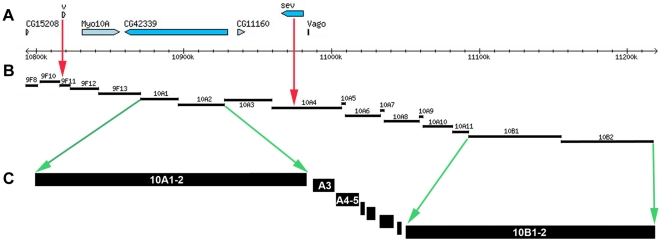
Comparison of extents of band/interbands in polytene chromosomes according to Flybase r5.25 (A, B) and to the data of this study (C). Physical DNA map is situated between 10792800 and 11220400 positions of the map of Flybase.

Data described here on mapping interbands and bands (as the distances between the borders of two neighboring interbands) provide a new and very good opportunity to map the band/interband positions on the cytological map of *Drosophila melanogaster* genome. Upon side-by-side comparison of band positions in the region studied (9F13 – 10B3) - obtained in the present work (Table 1) and those shown in FlyBase, - very little overlap is observed (Fig. 7A, B, C). Most of the discrepancies are found for the lengths and positions of bands and genes. For example, according to the FlyBase, *vermilion* gene maps to 9F11 and *sevenless* is annotated to belong to the non-existing band on the Bridges map - 10A4 (Fig. 7A, B, C). In fact, the correct position for *vermilion* is distal part of 10A1–2, some 25 kb away from its distal edge, and that of *sevenless* – in the proximal part of 10A1–2, - which has been recognized for over several decades [4], [55], [57], [58]. In the present work, where we mapped interbands, and then filled the regions in between as bands, we confirmed the localization of *vermilion* and *sevenless* genes within 26 kb from the distal edge and on the proximal side of 10A1–2, respectively (Fig. 7A, C). The reasons for these discrepancies most probably stem from the fact that sizes and positions of bands in FlyBase were determined not experimentally, rather they were calculated based on the data of V. Sorsa [68] for the DNA content in bands, which in turn were the result of computation. This approach has an error comparable with the size of one or two bands (http://flybase.org/static_pages/docs/refman/refman-G.html#G5). The precise mapping of the band/interband positions across the entire *Drosophila* genome using our approach is a subject of a separate work which is currently underway.

## Materials and Methods

### Cytological analysis of polytene chromosomes

Salivary gland polytene chromosome squashes were prepared for cytological electron microscopy analysis and examined as described earlier [69]. The 120–150 nm sections were cut using an LKB-IV (Sweden) ultratome and examined under a JEM-100C (JEOL, Japan) electron microscope at 80 kV.

### Fluorescence *in situ* hybridization (FISH)

Larvae were grown at 22°C in uncrowded vials on standard flyfood. Salivary glands were dissected in Ephrussi-Beadle solution, and then fixed in a 3∶1 mixture of ethanol and acetic acid for 30 minutes at −20°C, squashed in 45% acetic acid, snap-frozen in liquid nitrogen and stored in 70% ethanol at −20°C. For fluorescence *in situ* hybridization (FISH) on polytene chromosomes DNA probes were labeled with biotin-16-dUTP or digoxigenin-11-dUTP (Roche) in random-primed polymerase reaction with Klenow fragment [70]. Table 3 summarizes all the probes used in this study.

**Table 3:** Coordinates and descriptions of probes, selected for FISH mapping on polytene chromosomes in the region 9F13 – 10B3 (coordinates correspond to the version dm3 (r5.24), FlyBase).

Probes	Localization of probes on cytological map(See Results)	Description of probes	Primers	Coordinates of probe
CG1582	DNA of the interband 9F13/10A1–2 (arrow 1 on Fig. 2)	The fourth coding exon of the gene CG1582	5′-GCTTTTCCCTCGCCCAAGCG-3′; 5′-AAGAGGGCGGCATTGAGCGT-3′	10791234..10791994
spas_9F_10A	DNA of the interband 9F13/10A1–2 (arrow 1 on Fig. 2)	Intergenic fragment between genes *CG1582* and *CG15208*	5′-GGCGCGAAAGTGTGACCAGC-3′; 5′-GCTGGCAAGCGGGGCTGTAA-3′	10793537..10794176
CG15208	DNA of the interband 9F13/10A1–2 (arrow 1 on Fig. 2)	Coding exon of the gene *CG15208*	5′-GCTCTATTGCCGCTGGCTCC-3′; 5′-ACAGATGTCCGGGTGGGGTC-3′	10794863..10795492
Vago	DNA of the interband 10A1–2/10A3 (arrow 2 on Fig. 2)	The part of coding first exon, intron and parts of the second exon of the *Vago* gene	5′-GGTGGCAGCCAAGCGATTCC-3′; 5′-AATCTCGCCACGAGGGGGTG-3′	10983458..10983987
CG2076	The fragment of the interband 10A1–2/10A3 (arrow 2 on Fig. 2)	The second, third, and fourth coding exons, as well as the second, third and fourth introns of the *CG2076* gene	5′-TGGGCGCCCTGTGCTACTAC-3′; 5′-CATGGAGGCCAGACCTGCGA-3′	10984947..10985771
CG32668	Fragments of the interband 10A8–9/10B1–2 (arrow 3 on Fig. 2)	First coding exon of the *CG32668* gene	5′-CGACGATCTGTCCGCCTCGT-3′; 5′-CAAGGCATCGTGCGCTTCCG-3′	11050925..11051486
l(1)10Bb	Fragments of the intreband 10B1–2 10B3 (arrow 4 on Fig. 2)	Coding fragments of first and second exons and intron of the *l(1)10Bb* gene	5′-CAGTCGCAAACCACCGCCAG-3′; 5′-TCTAACCGGAGCAGCCGCGA-3′	11218702..11219201

Chromosomes were examined using epifluorescence optics (Olympus BX50 microscope) and photographed with CCD Olympus DP50.

### FISH combined with immunostaining of polytene chromosomes

Salivary glands were dissected in PBS containing 0.5% Tween-20. Glands were transferred into a drop of PBS containing 3.7% formaldehyde and 0.1% Triton X-100 for 40 sec. The solution was then replaced by a drop of 45% acetic acid with 3.7% formaldehyde for 1 min. Glands were squashed in 45% acetic acid and then frozen in liquid nitrogen. Spreads were dehydrated in 95% ethanol for 15 min and stored in 70% ethanol at −20°C for no longer than one week. FISH combined with immunostaining of polytene chromosomes was performed according to Lavrov et al. [71] with modifications described by Grimaud et al. and available from http://www.igh.cnrs.fr/equip/cavalli/Lab%20Protocols/FISH-Immuno_Grimaud.pdf. Anti-Chriz/CHRO [8] primary antiserum at a 1∶600 dilution was used. Rhodamine-labeled goat anti-rabbit IgG-specific conjugates (Abcam, 1∶200) were used as a secondary antibody. DNA probes were labeled with biotin-16-dUTP (Roche) as described above.

### Using the modENCODE Data on proteins

The data of modENCODE (http://www.modencode.org) project as well as some other papers were used. The data on them either are on corresponding page in GEO (http://www.ncbi.nlm.nih.gov/geo/), or in supplementary to the original papers (Table 4). We used two types of data from modEncode: Smoothed M-value enrichment profiles represented as histograms and Regions of significant enrichment. Protocols of data processing are described in corresponding section of modMine (http://intermine.modencode.org). Track for density of P- element insertion site was based on FlyBase, r5.32. We treated the data using the sliding window of 1 kb size with the step 500 b.p.

**Table 4:** Accession numbers of chromosome proteins.

№	Proteins and antibodies	Accession numbers (id)*
1	BEAF-32; BEAF-HB.S2	modENCODE_274
2	BEAF-32; BEAF-70.S2	modENCODE_922
3	Chriz (or Chromator); Chro(Chriz)WR.S2	modENCODE_279
4	Chriz (or Chromator); Chro(Chriz)BR.S2	modENCODE_278
14	RNApol II; RNA pol II(ALG).S2	modENCODE_329
16	Trx; Trx-C.S2	modENCODE_332
17	GAF; GAF.S2	modENCODE_285
18	BRE1; BRE1_Q2539.S2	modENCODE_923
21	NURF301; NURF301_Q2602.S2	modENCODE_947
22	WDS; WDS_Q2691.S2	modENCODE_953
32	H3K9acS10P (new lot); H3K9acS10P_(new_lot).S2	modENCODE_2660
39	ORC2; dORC2.S2	modENCODE_2753
40	ORC2; dORC2.BG3	modENCODE_2754
41	ORC2; dORC2.Kc	modENCODE_2755
43	Nucleosome_Density.S2	modENCODE_2506
44	30-State_Chromatin.S2	modENCODE_3365
45	30-State_Chromatin.BG3	modENCODE_3364
46	H3.3	GSE4091**
47	H1 dips	GSE16885
48	Five chromatin types	GSE22069
49	Lamin	GSM509085
50	D1	GSM550422
51	SUUR	GSM550486
52	Gene density	GSE16531
53	CATCH-IT	GSM494308
54	replication timing of S2 cells	GSM336376
55	replication timing of Kc cells	GSM336362
56	replication timing of Cl8 cells	GSM336363
57	DHSs in S2, Kc, BG3 cells	http://compbio.med.harvard.edu/flychromatin/data.html

*modENCODE data used (http://www.modencode.org/Genomes.shtml).

**GEO data used (http://www.ncbi.nlm.nih.gov/gds/).

For visualization of data we used UCSC Genome browser (http://genome.ucsc.edu). Custom scripts were used to adjust data in accordance with UCSC format.
