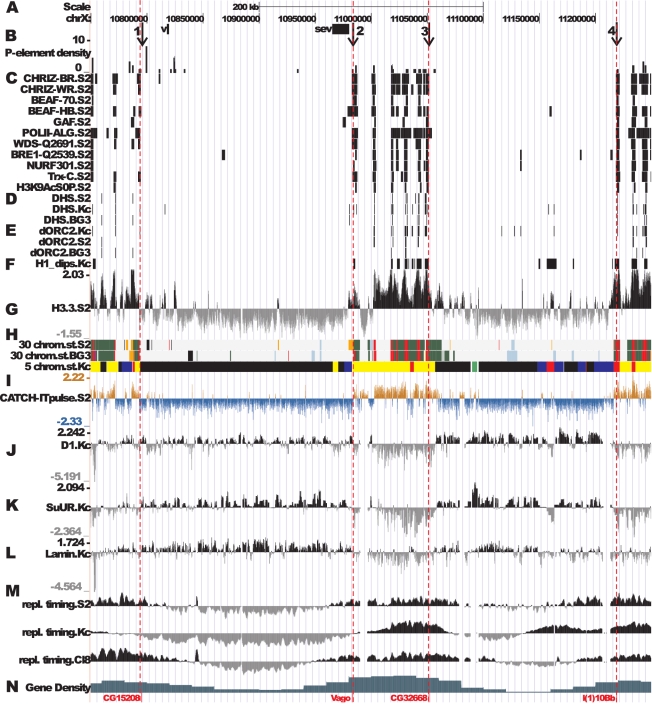# Correction: Identical Functional Organization of Nonpolytene and Polytene Chromosomes in *Drosophila melanogaster*

**DOI:** 10.1371/annotation/45b44e2a-c751-418b-bbb7-7023998abdfc

**Published:** 2011-11-15

**Authors:** Tatyana Yu. Vatolina, Lidiya V. Boldyreva, Olga V. Demakova, Sergey A. Demakov, Elena B. Kokoza, Valeriy F. Semeshin, Vladimir N. Babenko, Fedor P. Goncharov, Elena S. Belyaeva, Igor F. Zhimulev

There is an error in Figure 2. The correct Figure 2 can be seen here: 

**Figure:**